# Preparation of environmentally responsive PDA&DOX@LAC live drug carrier for synergistic tumor therapy

**DOI:** 10.1038/s41598-024-66966-2

**Published:** 2024-07-10

**Authors:** Lu Liu, Xuefen Zhao

**Affiliations:** 1 Northern Jiangsu People’s Hospital, Yangzhou, 225001 People’s Republic of China; 2 The Affiliated Huai’an Hospital of Xuzhou Medical University and The Second People’s Hospital of Huai’an, No. 62, Huaihai Road (S.), Huai’an, 223002 People’s Republic of China

**Keywords:** Polydopamine, Combination therapy, Environmental response, Precise release, Cancer, Drug discovery, Immunology, Materials science

## Abstract

The development of intelligent, environmentally responsive and biocompatible photothermal system holds significant importance for the photothermal combined therapy of tumors. In this study, inspired by *Lactobacillus* (LAC), we prepared a biomimetic nanoplatform PDA&DOX@LAC for tumor photothermal-chemotherapy by integrating the chemotherapeutic drug doxorubicin (DOX) with dopamine through oxidative polymerization to form polydopamine (PDA) on the surface of LAC. The PDA&DOX@LAC nanoplatform not only achieves precise and controlled release of DOX based on the slightly acidic microenvironment of tumor tissues, but also exhibits enzyme-like properties to alleviate tumor hypoxia. Under near-infrared light irradiation, it effectively induces photothermal ablation of tumor cells, enhances cellular uptake of DOX with increasing temperature, and thus efficiently inhibits tumor cell growth. Moreover, it is further confirmed in vivo experiments that photothermal therapy combined with PDA&DOX@LAC induces tumor cells apoptosis, releases tumor-associated antigens, which is engulfed by dendritic cells to activate cytotoxic T lymphocytes, thereby effectively suppressing tumor growth and prolonging the survival period of 4T1 tumor-bearing mice. Therefore, the PDA&DOX@LAC nanoplatform holds immense potential in precise tumor targeting as well as photothermal combined therapy and provides valuable insights and theoretical foundations for the development of novel tumor treatment strategies based on endogenous substances within the body.

## Introduction

Tumor remains one of the principal threats to human health and chemotherapy is still the primary clinical approach for its treatment^1,2^. While this method effectively suppresses tumor growth, it brings about significant toxic side effects to normal tissues and the immune system^3,4^. Therefore, there is a pressing need to develop a safer and more effective treatment modalities for tumor.

With the deeper understanding of tumor biology, researchers have observed that tumor tissues lack sufficient blood supply, rendering them unable to dissipate heat by regulating blood flow and velocity^5,6^. Consequently, tumor tissues become natural reservoirs for heat accumulation. In light of the monumental development of photothermal therapy for tumors, there is a need to mitigate the damage caused by singular photothermal therapy to surrounding normal cells and tissues. Therefore, researchers have taken a different approach which utilizes near-infrared light (NIR) to induce localized heat generation within tumor tissues, thereby triggering apoptosis in tumor cells^7,8^. Despite the advantages of this method such as low invasiveness, high selectivity and temporal-spatial control, the effective penetration of NIR is limited, making it challenging to eradicate deep-seated tumor cells and hindering its clinical application^9,10^. Therefore, there is an urgent need for safer and more effective methods for treating tumors.

With the widespread development and application of photosensitizers and photothermal materials, the photothermal-chemotherapy combination therapy effectively overcomes the shortcoming of individual treatments^11^. Especially, environmentally responsive nanoplatform prepared based on tumor microenvironment not only achieves precise and controllable release of chemotherapeutic drugs in tumor tissues, but also effectively improves the tumor tissue microenvironment, thereby achieving a cascade effect in tumor treatment^12,13^. Currently, extensively-developed and primarily-utilized nanoplatform is consist of exogenous inorganic and organic compounds. However, due to their non-degradability and suboptimal biological metabolism, these carriers inevitably bring unnecessary toxic side effects to normal tissues and organs, hindering the clinical implementation of photothermal-chemotherapy combination therapy^14,15^.

The development of photothermal nanomaterials based on endogenous substances can effectively reduce the body's immune response, minimize damage to normal tissues and better meet the requirements for future clinical translation^16^. Currently, widely developed and utilized endogenous photothermal materials mainly include dopamine, serotonin and adrenaline^17^. Among them, dopamine, as one of the important neurotransmitters in the body, plays a crucial role in regulating the development of the nervous system and maintaining pleasure and happiness in humans^18^. Furthermore, as a small molecule catecholamine in the human body, dopamine can undergo oxidative polymerization in an alkaline environment and form size-controllable polydopamine (PDA) nanoparticles^19^. The prepared polydopamine nanoparticles exhibits superior photothermal performance in the near-infrared region. At the same time, their unique conjugated molecular structure and special adhesion properties make them highly suitable for loading chemotherapeutic drugs^20^. More importantly, the surface of polydopamine nanoparticles contains abundant amino and hydroxyl groups, which can facilitate surface modification to impart more biological properties, enabling tumor combination therapy based on photothermal effects^21^. Although the biocompatibility of photothermal nanoplatform have been effectively addressed, similar to most photothermal nanoparticles, polydopamine nanoparticles cannot achieve targeted delivery. Moreover, the central region of tumor tissues is severely hypoxic, resulting in the ineffective killing of tumor cells in the central region of tumor tissues by most antitumor drugs and photothermal nanoparticles^22^.

**Scheme 1:**
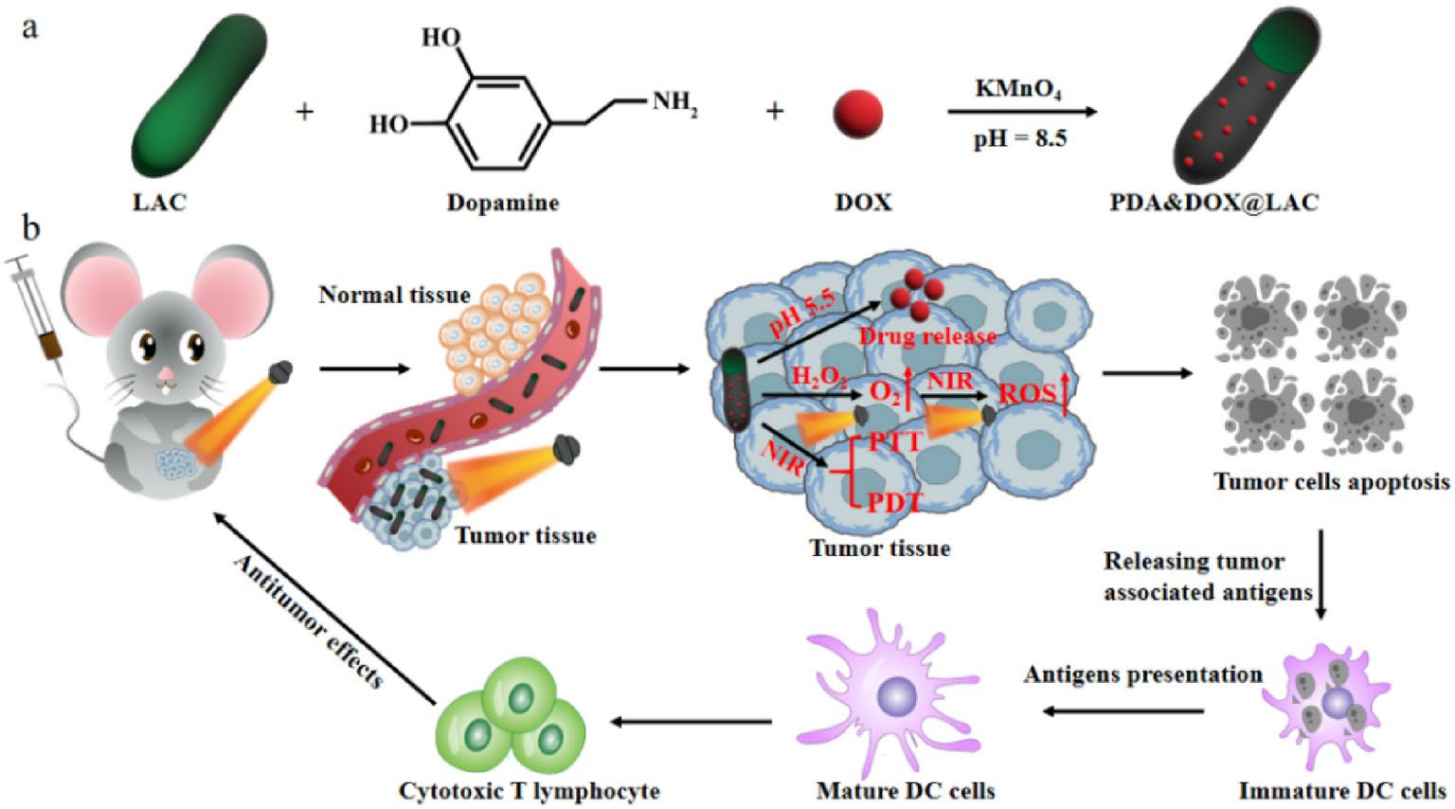
The preparation of PDA&DOX@LAC nanoplatform for tumor synergistic therapy. (**a**) Construction of a PDA&DOX@LAC nanoplatform. (**b**) Proposed action mechanism of PDA&DOX@LAC nanoplatform in a mouse tumor model.

To sum up, in this study, as a living drug carrier, *Lactobacillus* (LAC) integrates the chemotherapeutic drug doxorubicin (DOX) with dopamine through oxidative polymerization to form polydopamine on the surface of LAC and prepares an environmentally responsive PDA&DOX@LAC biomimetic therapeutic platform for photothermal-chemotherapy combined treatment of tumors. As illustrated in Scheme 1a, the one-step preparation process of the PDA&DOX@LAC nanoplatform is gentle, with negligible impact on the biological properties of LAC. Upon intravenous injection into mice, the prepared PDA&DOX@LAC nanoplatform utilizes the anaerobic property of LAC to deliver the antitumor drug DOX to tumor tissues and achieves precise and controlled release of DOX based on its protonation propensity under acidic conditions. While preparing the PDA&DOX@LAC nanoplatform, doped Mn ions display peroxidase-like properties, which enable them to react with the high concentration of hydrogen peroxide in tumor tissues, leading to the production of oxygen. This process effectively alleviates the hypoxic condition within the tumor tissues. Upon being exposed under NIR irradiation, the PDA&DOX@LAC nanoplatform converts light energy into heat and generates a large amount of reactive oxygen species, inducing apoptosis of tumor cells and releasing tumor-associated antigens. These antigens are taken up by dendritic cells and stimulate infiltration of cytotoxic T cells, which in turn kill tumor cells and inhibit tumor growth (Scheme 1b). Therefore, the PDA&DOX@LAC nanoplatform has been developed in a simpler and more effective manner. It can not only serve as an efficient live drug carrier for chemotherapy with tumor tissue-targeted delivery, but also exhibits peroxidase-like activity to effectively improve the tumor hypoxic microenvironment, thus providing a theoretical basis and design concept for the development of novel tumor treatment platforms in the future.

## Materials and reagents

Dopamine, reduced glutathione (GSH), hydrogen peroxide (H_2_O_2_), 4’,6-diamidino-2-phenylindole (DAPI), doxorubicin (DOX), [Ru(dpp)_3_]Cl_2_ (RDPP), 2′,7′-dichlorofluorescein diacetate (DCFH-DA), 1,3-diphenylisobenzofuran (DPBF), cell counting kit-8 (CCK-8), MRS broth and sodium hydroxide (NaOH) were procured from Aladdin (Shanghai, China). Dulbecco’s modified eagle medium (DMEM), RPMI Medium Modified (RPMI-1640), fetal bovine serum (FBS), phosphate-buffered saline (PBS) and streptomycin and penicillin were obtained from Gibco (Shanghai, China). All chemicals were utilized as received without additional purification. Deionized water was used throughout the experiments.

### Apparatus and procedures

Transmission electron microscopy (TEM) imaging was conducted using a JEOL 2100 transmission electron microscope. Particle size and ζ-potential measurements were performed using a NanoBrook Omni (Brookhaven, USA). Fourier transform infrared (FTIR) spectra were obtained with a Nicolet Nexus 470 spectrometer (USA). X-ray photoelectron spectroscopy (XPS) analysis of PDA&DOX@LAC was carried out using an XPS spectrometer (Thermo Kalpha). Near-infrared light at 808 nm was delivered using a fiber-coupled NIR laser (MDL-N-808 nm-10W, Beijing Laserwave OptoElectronics Technology Co., Ltd., Beijing, China). An infrared thermal camera (HT-19, Guangzhou, China) was employed to monitor temperature changes and capture infrared thermal images.

### The preparation of PDA&DOX@LAC

The synthesis procedure of the PDA&DOX@LAC nanoplatform followed established protocols with slight adaptations^23,24^. Initially, 1 × 10^9^ Lactobacillus cells were dispersed in 10 mL of pH 8.0 PBS buffer. Subsequently, dopamine (1 mg/mL), doxorubicin (1 mg/mL) and 0.5 mL of KMnO_4_ (1 mg/mL) were introduced into the solution according to the prescribed method. The mixture was then stirred at room temperature for 30 min and the precipitate was collected via centrifugation (2000 rpm, 5 min) and subsequent washed with PBS for three times. Finally, the samples were freeze-dried under vacuum conditions. The obtainted PDA&DOX@LAC nanoplatform was stored at 4 ℃ for future applications.

### The release behavior of DOX from PDA&DOX@LAC

To assess the release kinetics of DOX from the PDA&DOX@LAC nanoplatform under various conditions, 1 × 10^7^ CFU of PDA&DOX@LAC nanoplatform was separately introduced into (a) PBS, (b) pH = 5.5 solution, (c) H_2_O_2_ (30 μM) solution, (d) 10 mM GSH solution and (e) a simulated tumor microenvironment (pH 5.5, 10 mM GSH, and 30 μM H_2_O_2_)^25^. Subsequently, at predetermined time intervals, samples were withdrawn from each mixture and the absorbance of DOX at 490 nm was measured using UV–vis spectroscopy to characterize the release profile of DOX.

### The measurement of photothermal effects

PDA&DOX@LAC nanoplatform was resuspended in 200 µL PBS and irradiated with 808 nm NIR (1.5, 1.75, and 2.0 W/cm^2^) at different powers for different time intervals at room temperature. PDA&DOX@LAC was configured at 100 μg/mL and 200 μg/mL (the number of LACs was approximately 5 × 10^6^ and 1 × 10^7^) and temperature changes were recorded at any time using an infrared thermal imaging instrument. The photothermal conversion efficiency (η) of PDA&DOX@LAC nanoplatform was calculated by the following Eq. ^26,27^:

1 $$ \theta = ({\text{T}} - {\text{T}}_{\min } ){/}({\text{T}}_{\max } - T_{\min } ) $$

2 $$ {\text{hs}} = m*{\text{C}}_{{\text{p}}} {/}\zeta {\text{s}} $$

3 $$ \eta = {\text{hs}}(\Delta {\text{T}}_{{\max ,{\text{mix}}}} - \Delta {\text{T}}_{{\max ,{\text{H}}2{\text{O}}}} ){\text{/I}}(1 - 10^{{ - {\text{A}}808}} ) $$

θ defined the dimensionless driving force temperature, m was 2 × 10^−4^ kg, Cp was 4.2 × 10^3^ J/(kg·℃), ζ is the slope of T and − Inθ, ΔTmax,mix is the maximum temperature change of PDA&DOX@LAC, ΔTmax,H_2_O is the maximum temperature change of water. I is the laser power. Where A808 is the absorbance of PDA&DOX@LAC nanoplatform at the wavelength of 808 nm in aqueous solution.

### Cell culture

4T1 cells (murine breast cancer cells), GES-1 cells (human gastric mucosa epithelial cells) and LAC (Lactobacillus) were procured from SUNNCELL Biological Technology Co., Ltd. The cells were cultured in RPMI Medium Modified (RPMI-1640) supplemented with 10% heat-inactivated FBS and 1% penicillin–streptomycin. Cell cultures were maintained at 37 °C in a 5% CO_2_ atmosphere and were approved for use by the Northern Jiangsu People’s Hospital.

### The evaluation of O_2_ generation ability from the PDA&DOX@LAC

The oxygen generation capability of PDA&DOX@LAC nanoplatform was assessed by dissolved oxygen meter (JPBJ-608, INESL, Shanghai, China)^28^. Initially, 10 mL of PDA&DOX@LAC dispersion solution (200 µg/mL) was added to a 20 mL beaker. Subsequently, H_2_O_2_ (30 µM) was introduced into the PDA&DOX@LAC dispersion solution. The dissolved oxygen meter probe was then inserted into the dispersion of PDA&DOX@LAC and H_2_O_2_ mix dispersion solution to monitor real-time changes in oxygen concentration. PBS solution, H_2_O_2_ solution and PDA&DOX@LAC dispersion served as control experiments.

[Ru(dpp)_3_]Cl_2_ (RDPP) serves as a fluorescent probe for dissolved oxygen detection and its fluorescence signal is quenched in the presence of oxygen^29^. Thus, 2 mg of PDA&DOX@LAC nanoplatform was added to 20 mL PBS solution containing 30 µM H_2_O_2_ and 10 µM RDPP. Fluorescence measurements were taken at predetermined time points by fluorescence spectrometer to detect oxygen concentration in the dispersion of PDA&DOX@LAC. Additionally, 10 µM RDPP fluorescent dye was separately added to 20 mL PBS solution, PBS solution containing H_2_O_2_ (30 µM H_2_O_2_), and PDA&DOX@LAC dispersion (200 µg/mL) as control groups to analyze oxygen concentration in the above solutions.

4T1 cells were co-incubated with PDA&DOX@LAC, and the oxygen generation capability within the cells was assessed by observing the intensity of RDPP fluorescence signals using fluorescence microscopy. The specific steps were as follows: Initially, 4T1 cells (1 × 10^7^) were seeded into cell culture dishes and co-incubated with 10 µM RDPP for 4 h. Then, H_2_O_2_ (30 µM), PDA&DOX@LAC (200 µg/mL), and PDA&DOX@LAC (200 µg/mL) + H_2_O_2_ (30 µM) were separately added to the aforementioned culture, then cultured for 4 h and washed with PBS several times. Finally, fluorescence imaging of 4T1 cells in each treatment group was observed using fluorescence microscopy^30^.

### The detection of extracellular and intracellular ROS

1,3-diphenylisobenzofuran (DPBF) serves as a chemical probe for monitoring extracellular reactive oxygen species (ROS) and exhibits decreased absorbance when exposing to ROS environments^31,32^. Initially, PDA&DOX@LAC (200 μg/mL) was added to a mixture of DPBF (1 mM) and H_2_O_2_ (30 μM) and then irradiated at 808 nm NIR for 10 min. UV–vis spectroscopy was then utilized to detect the UV–visible absorption peaks of DPBF at predetermined time points. DPBF in H_2_O_2_ solution (30 μM), dispersed solution of PDA&DOX@LAC (200 μg/mL) + H_2_O_2_ (30 μM), PDA&DOX@LAC (200 μg/mL) + NIR, and UV–visible absorption peaks in PDA&DOX@LAC served as control groups.

Additionally, the intracellular levels of ROS were assessed using the cell-permeable probe 2',7'-dichlorofluorescein diacetate (DCFH-DA)^33^. DCFH-DA does not exhibit any fluorescence signal under normal conditions; however, in the presence of abundant ROS within cells, DCFH-DA is rapidly oxidized to yield green fluorescence of 2,7-dichlorofluorescein (DCF)^34^. To further validate whether co-incubation of PDA&DOX@LAC with 4T1 cells could induce ROS generation under 808 nm NIR irradiation, 4T1 cells (1 × 10^7^) were seeded into 6-well plates and subjected to different treatments (PDA&DOX@LAC, H_2_O_2_, NIR, PDA&DOX@LAC + H_2_O_2_, PDA&DOX@LAC + NIR, and PDA&DOX@LAC + NIR + H_2_O_2_). After 6 h, the cells were exposed to NIR radiation of 2.0 W/cm^2^ for 10 min, and then the medium was removed and replaced with fresh medium containing DCFH-DA. After 30 min, the medium was discarded and the cells were washed three times with PBS before fluorescence signals within 4T1 cells were observed using fluorescence microscopy.

### The temperature regulates cell membrane permeability

Different concentrations of PDA&DOX@LAC (100 μg/mL and 200 μg/mL) were co-incubated with 4T1 cells and placed in a cell culture incubator (37 °C, 5% CO_2_). After 6 h, they were subjected to NIR irradiation at different power densities (1.5 and 2.0 W/cm^2^) for 10 min, followed by a incubation period of 3 h. Subsequently, the culture medium was discarded and the cells were washed three times with PBS. Then, 1 mL of 4% paraformaldehyde was added to each cell culture dish for fixation for 15 min. After discarding the fixative, 1 mL of DAPI staining solution was added to each dish and gently shaken on a horizontal shaker. After 15 min, the DAPI staining solution was removed and the cells were washed three times with PBS. 500 μL PBS was then added, and the fluorescence intensity of 4T1 cells and GES-1 cells was observed using confocal fluorescence microscopy^35,36^.

### In vitro cytotoxicity

The cytotoxicity of PDA&DOX@LAC combined with NIR was evaluated using the CCK-8 assay. 1 × 10^4^ 4T1 cells and GES-1 cells were seeded into 96-well plates and incubated for 12 h. Subsequently, different concentrations of PDA&DOX@LAC, DOX, PDA, and PDA&DOX nanoparticles were added to each well and co-incubated with the cells for 6 h, followed by irradiation under 808 nm NIR (2.0 W/cm^2^, 10 min). Untreated cells were used as a control group. After 24 h, the cells were washed several times with PBS, and then 10 µl of CCK-8 reagent was added to each well and further incubated for 3 h. The absorbance values (OD) of each well in the 96-well plate were measured using a microplate reader at an excitation wavelength of 450 nm^37,38^.

### Animal welfare

In this study, the animal experiment was conducted in accordance with the guidelines outlined in the Animal Management Rules of the ARRIVE guidelines. Healthy female C57BL/6 mice (number: NO.202459791), aged 6 to 8 weeks, were procured from the Model Animal Genetics Research Center of Yangzhou University (Yangzhou, People’s Republic of China). Every effort was made to minimize animal suffering and reduce the number of animals used in the study.

### The construction and treatment of 4T1 mouse subcutaneous xenografts model

Male Balb/c mice were subcutaneously injected with 0.2 mL of 4T1 cells (5 × 10^7^) in the groin area to establish the 4T1 subcutaneous tumor model^39,40^. When the tumor volume reached approximately 100 mm^3^, the 4T1 tumor-bearing mice were randomly divided into 6 groups (n = 6/group): PBS, PBS + NIR, DOX (4 mg/kg), PDA (4 mg/kg) + NIR, PDA&DOX (4 mg/kg) + NIR, and PDA&DOX@LAC + NIR groups. The aforementioned drugs were administered intravenously via the tail vein to the tumor-bearing mice. After 24 h post-injection, NIR irradiation at a power density of 2.0 W/cm^2^ was applied for 10 min. Injections were administered every 3 d, with real-time monitoring of each mouse's body weight and tumor volume. Tumor volume was calculated using the formula: 0.5 × width^2^ × length. Subsequently, tumor tissues and major organs (heart, liver, spleen, lungs, and kidneys) were fixed in 4% formalin buffer and embedded in paraffin. Specimens underwent hematoxylin–eosin (H&E) staining and Terminal Deoxynucleotidyl Transferase Dutp Nick End Labeling (TUNEL) staining.

### Histological analysis

After 15 d, all mice were euthanized using cervical dislocation, and the major organs (heart, liver, spleen, lungs, and kidneys) along with tumor tissues of the 4T1 tumor-bearing mice were collected for histological analysis. The collected tissues were fixed in 4% formalin and embedded in paraffin. Tissue sections were prepared and stained with H&E staining. Tumor tissues were also subjected to TUNEL staining. The prepared tissue sections were placed on a slide scanner to capture tissue images, which were then evaluated by experienced pathologists^40,41^.

### The detection of mouse blood samples

Before treatment, blood tests were conducted on 4T1 tumor-bearing mice in the PDA&DOX@LAC + NIR group. Subsequently, PDA&DOX@LAC (4 mg/kg) was injected intravenously via the tail vein into the 4T1 tumor-bearing mice. Blood samples were collected from the mice on days 1, 7, and 14 of treatment to evaluate hematological and biochemical parameters and assess the biocompatibility of PDA&DOX@LAC^42^.

### Tumor dendritic cells and infiltrating T cells analysis

After treatment, tumor tissues were collected from each mouse in every treatment group. These tissues were minced and digested using Dulbecco's Modified Eagle's Medium (DMEM) supplemented with hyaluronidase (100 μg/mL), DNase I (100 μg/mL), 10% fetal bovine serum (FBS), and collagenase type IV (1 mg/mL) under continuous agitation (200 rpm) in an incubator at 37 °C. Following digestion, the cells were filtered through a nylon mesh (500 mesh) and then centrifuged at 500 g for 5 min. The resulting cell pellet was subjected to red blood cell lysis and purified using a 40% Percoll (GE) solution. Dendritic cells isolated from the extracted cell population were stained with anti-mouse CD11c, CD80, and CD86 antibodies for flow cytometric analysis. Additionally, cytotoxic T lymphocytes present in the single-cell suspension obtained from 4T1 xenograft tumors were stained with anti-mouse CD3, CD4, and CD8a antibodies for subsequent flow cytometric analysis^43,44^.

### Analysis of tumor dendritic cells and infiltrating T cells

After treatment, tumor tissues were collected from each mouse of each treatment group and continuously shaken in an oven at 37 ℃ (200 rpm, 40 min). Then, they were chopped and digested with Dulbecco modified Dulbecco medium (DMEM) containing hyaluronidase (100 μg/mL), deoxyribonuclease I (100 μg/mL), 10% fetal bovine serum (FBS) and collagenase IV (1 mg/mL). Subsequently, the digested cells were filtered with a nylon mesh (500 mesh), collected by centrifugation at 500 g for 5 min and then purified by using erythrocyte lysis and 40% Percoll (GE) solution. The extracted dendritic cells were collected and stained with anti-mouse CD11c, anti-mouse CD80 and anti-mouse CD86 for flow cytometry analysis. In addition, cytotoxic T lymphocytes in single cell suspension were collected from 4T1 xenograft tumors and stained with anti-mouse CD3, anti-mouse CD4 and anti-mouse CD8a for flow cytometry analysis^45,46^.

### Statistical analysis

The experimental data were analyzed by SPSS (17.0) software, OriginPro (2017) and Graphad Prism5. All data were presented by mean ± standard deviation. Student’s t-test was used to analyze Two-group comparison and the data from more than three groups were compared and analyzed by one-way analysis of variance (ANOVA). The *p* value of < 0.05 was considered statistically significant. ‘*** ’ means *p* < 0.001, ‘**’ means *p* < 0.01, and ‘*’ means *p* < 0.05.

### Ethics approval and consent to participate

All experimental procedures were approved by the Institutional Animal Care and Use Committee of Northern Jiangsu People’s Hospital (no. UJS-IACUC-AP-20190314002).

## Results and discussion

### The preparation and characterization of PDA&DOX@LAC nanosystem

**Figure 1:**
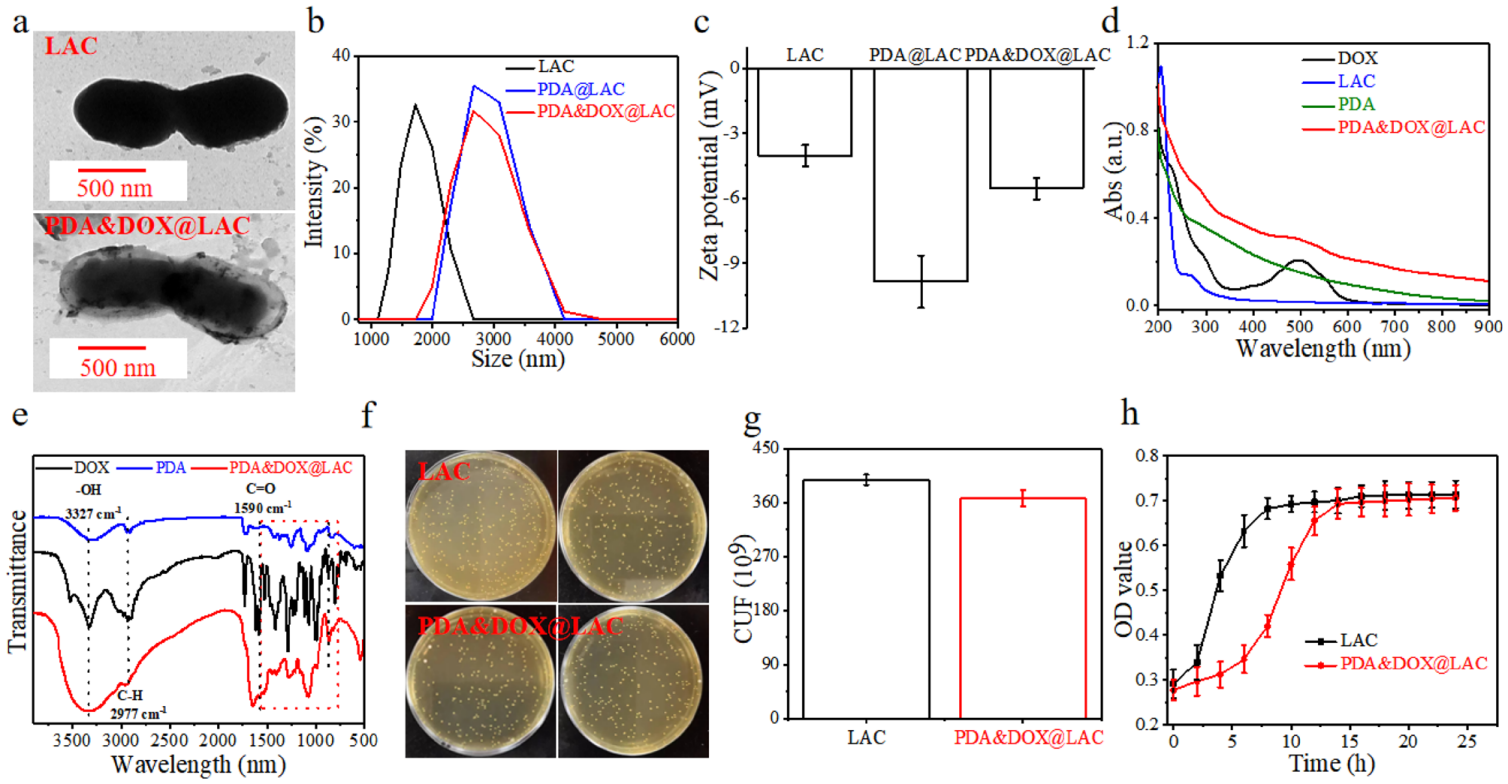
Characterization of PDA&DOX@LAC nanosystem. (**a**) Representative TEM images of LAC and PDA&DOX@LAC. Scale bar: 500 nm. (**b**) Size distribution of LAC, PDA@LAC and PDA&DOX@LAC. (**c**) Zeta potential of LAC, PDA@LAC and PDA&DOX@LAC. (**d**) UV/Vis spectra of DOX, LAC, PDA and PDA&DOX@LAC. (**e**) FTIR spectra of DOX, PDA and PDA&DOX@LAC. (**f**) Bacteria clones and bacterial viability of LAC and PDA&DOX@LAC after incubation for 48 h at 37 °C. (**g**) The corresponding number of viable bacteria of LAC and PDA&DOX@LAC. (**h**) The corresponding OD value of LAC and PDA&DOX@LAC at different time point.

Since W. Busch and W. Coley made pioneering works of utilizing bacteria in tumor therapy, bacterial-mediated tumor treatment has been extensively explored and utilized^47,48^. With advancements in biotechnology, materials science, and immunology, bacteria have been genetically engineered to serve as specific immunotherapeutic agents, which is capable of ameliorating the tumor immunosuppressive microenvironment and enhancing antitumor immune responses^49,50^. However, the safety concerns associated with genetically engineered bacteria, including the risk of gene transfer and mutation, have hindered their widespread application as therapeutic vectors^51^. In recent years, inspired by advances in materials science, researchers have employed methods such as covalent coupling, supramolecular interactions and physical encapsulation to modify bacteria, effectively enhancing the accumulation and retention of therapeutic drugs at tumor sites and imparting additional biological functions^52^. Motivated by these strategies, this study utilized the oxidative polymerization property of dopamine in alkaline environments to coat chemotherapeutic drug DOX onto the surface of LAC. TEM images demonstrated the successful modification of LAC surface with dopamine and DOX at concentrations of 1 mg/mL, resulting in the formation of uniform nanoparticles with a core–shell structure, which are PDA&DOX@LAC nanosystem (Fig. 1a). Dynamic light scattering results indicated an increase in average particle size from 1803 ± 158 nm to 2586 ± 312 nm upon PDA loading (Fig. 1b). Additionally, electrochemical potential observations revealed a shift in potential from − 3.89 ± 0.69 mV (LAC) to − 10.2 ± 1.26 mV (PDA@LAC) after polydopamine coating and a further elevation to − 5.89 ± 0.93 mV after DOX was successfully loaded on the surface of PDA&DOX@LAC nano-system(Fig. 1c). To further confirm the successful loading of DOX into the PDA&DOX@LAC nanocarrier, the results of UV/Vis spectroscopic showed a significant increase in absorbance in the suspension of PDA&DOX@LAC nanosystem and the absorption peak position matched with that of DOX (Fig. 1d). Furthermore, the results of fourier transform infrared (FTIR) spectroscopy revealed similar vibrational characteristic peaks at 3327 cm^-1^, 2977 cm^-1^, and 1590 cm^-1^ for DOX, PDA, and PDA&DOX@LAC nanosystem, corresponding to –OH, C-H, and C = O stretching vibrations, respectively. Moreover, DOX and PDA&DOX@LAC nanosystem exhibited similar absorption peaks between 900–1500 cm^-1^, which confirmed the successful preparation of the PDA&DOX@LAC nanosystem (Fig. 1e). Subsequently, the biocompatibility of the PDA&DOX@LAC nanosystem during the preparation process was further validated. Colony counting showed that the survival rate of LAC after encapsulation remained around 94.5% (Fig. 1f and 1g). Finally, the impact of the polydopamine shell on bacterial growth was evaluated through optical density (OD) measurements, revealing that the polydopamine coating effectively delayed LAC growth without affecting its viability (Fig. 1h). In conclusion, a core–shell structured PDA&DOX@LAC nanosystem was successfully prepared.

**Figure 2:**
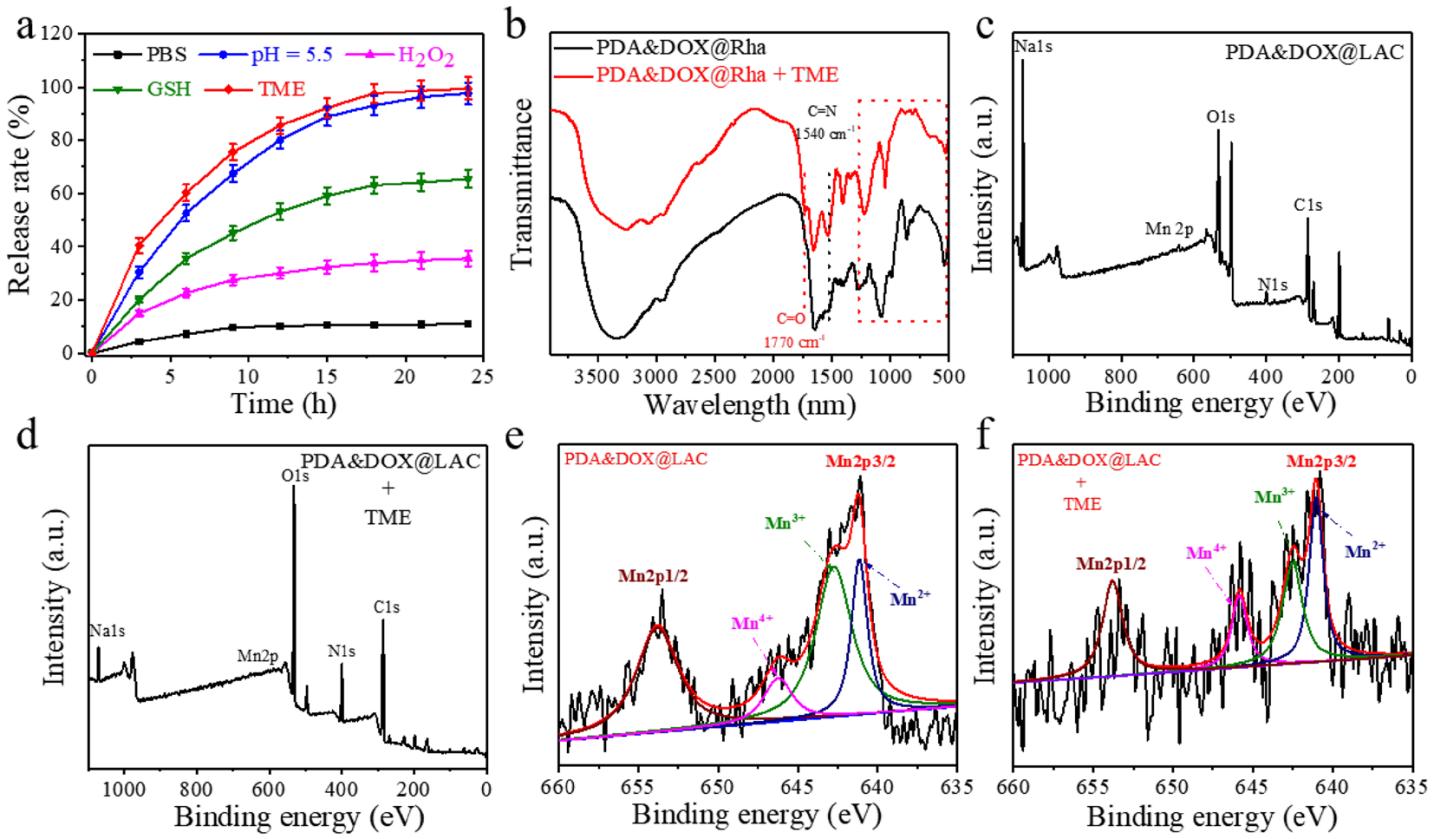
(**a**) In vitro profiles of DOX release from PDA&DOX@LAC nanosystem under conditions of PBS, pH = 5.5, H_2_O_2_, GSH and simulated TME. (**b**) FTIR spectra of PDA&DOX@LAC and PDA&DOX@LAC immersed into simulated TME. (**c**) XPS of PDA&DOX@LAC nanosystem. (**d**) XPS of PDA&DOX@LAC nanosystem immersed into simulated TME. (**e**) Narrow x-ray photoelectron spectroscopy scan spectra of Mn2p in PDA&DOX@LAC nanosystem. (**f**) Narrow x-ray photoelectron spectroscopy scan spectra of Mn2p in PDA&DOX@LAC nanosystem immersed into simulated TME.

In order to evaluate the environmentally responsive release performance of the PDA&DOX@LAC nanosystem, we conducted X-ray photoelectron spectroscopy (XPS) analysis. The results revealed that the PDA&DOX@LAC nanosystem was composed of Na, Mn, O, N, and C elements, with manganese ions primarily existing in the forms of Mn^3+^ and Mn^4+^ (Fig. 2c and 2e). Subsequently, the PDA&DOX@LAC nanosystem was immersed in five different simulated solutions: PBS, pH = 5.5, 30 µM H_2_O_2_, 10 mM GSH and a simulated tumor microenvironment (TME) (pH = 5.5, 30 µM H_2_O_2_, 10 mM GSH). As depicted in Fig. 2a, only approximately 15% of DOX was released in the normal physiological environment (PBS), mainly due to the flow of the solution causing DOX release from the surface of the PDA&DOX@LAC nanosystem. However, in the H_2_O_2_ solution, around 22.3% of DOX released from the surface of the PDA&DOX@LAC nanosystem. This release was primarily attributed to the accumulation of H_2_O_2_ on the surface of the PDA&DOX@LAC nanosystem, where H_2_O_2_ can oxidize the phenolic hydroxyl groups on polydopamine and weaken the hydrogen bonding interactions between drugs and polydopamine, thus causing DOX releasing^53^. When the PDA&DOX@LAC nanosystem was immersed in the GSH solution, nearly 60% of DOX released. This significant release was attributed to the redox reaction between GSH and the PDA&DOX@LAC nanosystem^54^. Although the elemental composition of PDA&DOX@LAC remained unchanged, a large amount of Mn^3+^ and Mn^4+^ reduced to Mn^2+^, causing structural changes in the PDA&DOX@LAC nanosystem and subsequently leading to DOX releasing (Fig. 2d and 2f). However, when the PDA&DOX@LAC nanosystem was immersed in an acidic environment, the release rate approached 100% after 24 h. The results of fourier-transform infrared spectroscopy (FTIR) spectra shown that while the absorption peak positions of PDA&DOX@LAC and PDA&DOX@LAC + TME were the same, the absorption peak of DOX between 900 and 1500 cm^−1^ in PDA&DOX@LAC + TME was weakened (Fig. 2b). This weakening was mainly attributed to the protonation of DOX in the acidic environment leads to its release from the surface of the PDA&DOX@LAC nanosystem^55,56^. When the PDA&DOX@LAC nanosystem was immersed in an in vitro simulated TME, nearly 100% of DOX released within 18 h. The rapid release of DOX was attributed to the combined effects of multiple factors. Thus, the prepared PDA&DOX@LAC nanosystem exhibited environmentally responsive release performance.

### Enzyme-like property of PDA&DOX@LAC nanosystem

Due to the inclusion of manganese ions in the PDA&DOX@LAC nanosystem, which possess catalase-like activity, they can react with the high concentration of H_2_O_2_ in tumor tissues and generate oxygen as well as hydroxyl radicals. This process effectively alleviates the hypoxic microenvironment of tumor tissues and the produced hydroxyl radicals further damage tumor cells^57,58^.

**Figure 3:**
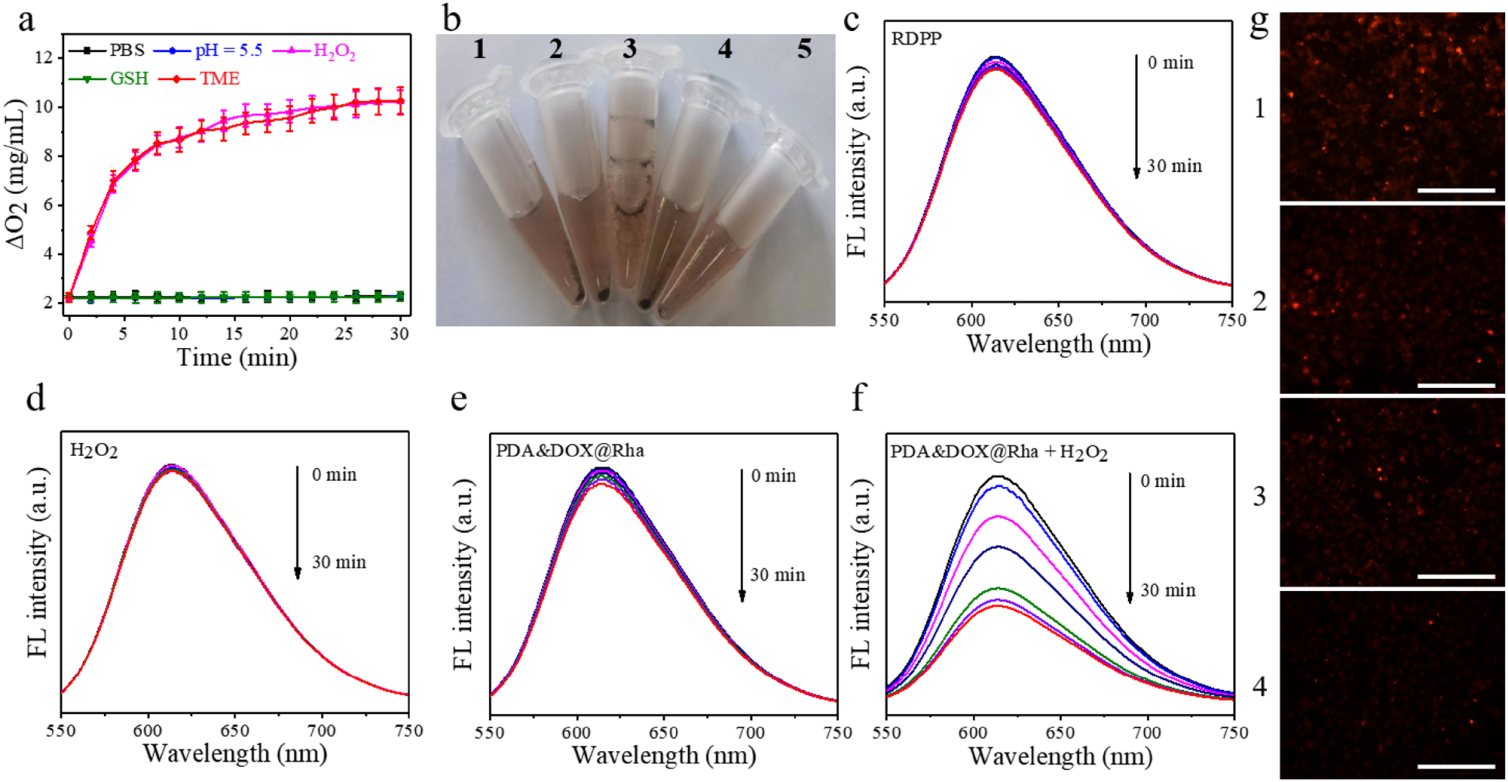
(**a**) H_2_O_2_-triggered O_2_ generation in different solutions. (**b**) Photograph of H_2_O_2_-triggered O_2_ generation in different solutions. (PDA&DOX@LAC nanosystem immersed into 1: PBS, 2: pH = 5.5, 3: H_2_O_2_, 4: GSH, 5: TME). Fluorescence spectra of RDPP in PBS (**c**), H_2_O_2_ (**d**), PDA&DOX@LAC nanosystem (**e**,**f**) PDA&DOX@LAC nanosystem + H_2_O_2_. (**g**) CLSM images of RDPP in 4T1 cells without any treatments (1), and treated with H_2_O_2_ (2), PDA&DOX@LAC nanosystem and PDA&DOX@LAC nanosystem + H_2_O_2_ (4). Scale bars: 300 μm. Each sample was repeated three times.

In order to investigate the oxygen-generating capacity of the PDA&DOX@LAC nanosystem in response to H_2_O_2_, the nanosystem was immersed in five different simulated solutions: PBS, pH = 5.5, 30 µM H_2_O_2_, 10 mM GSH, and a simulated TME. The oxygen content in each solution was monitored in real-time by a dissolved oxygen meter. Over time, as the PDA&DOX@LAC nanosystem was immersed in the H_2_O_2_ solution and TME, the oxygen content gradually increased. After 26 min, the oxygen content in these two solutions respectively reached 9.87 mg/mL and 9.93 mg/mL, while the oxygen content in the other solutions remained relatively unchanged (Fig. 3a). Photographs taken of each reaction system revealed a greater number of bubbles when the PDA&DOX@LAC nanosystem was immersed in the solution containing H_2_O_2_ (Fig. 3b). RDPP, serving as a typical oxygen fluorescence probe, is oxidized in the presence of oxygen and leads to quenching of its fluorescence signal. As shown in Figs. 3c–f, when RDPP was immersed in a mixture of PDA&DOX@LAC and 30 μM H_2_O_2_ solution, the fluorescence signal of RDPP gradually weakened. However, in the solution containing RDPP, H_2_O_2_, and PDA&DOX@LAC, there was no significant change in the RDPP fluorescence signal. This result indicates that the dispersion of PDA&DOX@LAC in the H_2_O_2_ solution generated oxygen, causing the oxidation of RDPP and subsequent reduction in its fluorescence signal. In an in vitro simulated high-concentration H_2_O_2_ environment, PDA&DOX@LAC generated a significant amount of oxygen, prompting further the evaluation of intracellular oxygen production. Subsequently, PDA&DOX@LAC was co-incubated with 4T1 cells to assess intratumoral oxygen generation. As shown in Fig. 3g, when 4T1 cells were co-incubated with PDA&DOX@LAC, the RDPP fluorescence signal exhibited noticeable quenching under fluorescence microscopy. Conversely, when 4T1 cells were co-incubated with PDA&DOX@LAC and H_2_O_2_, the red fluorescence signal was barely detected. In comparison, strong red fluorescence signals were observed when 4T1 cells were solely incubated with RDPP or co-incubated with H_2_O_2_. These results suggested that PDA&DOX@LAC possesses catalase-like properties and is capable of generating oxygen through its reaction with H_2_O_2_.

### Photothermal properties of PDA&DOX@LAC nanosystem

**Figure 4:**
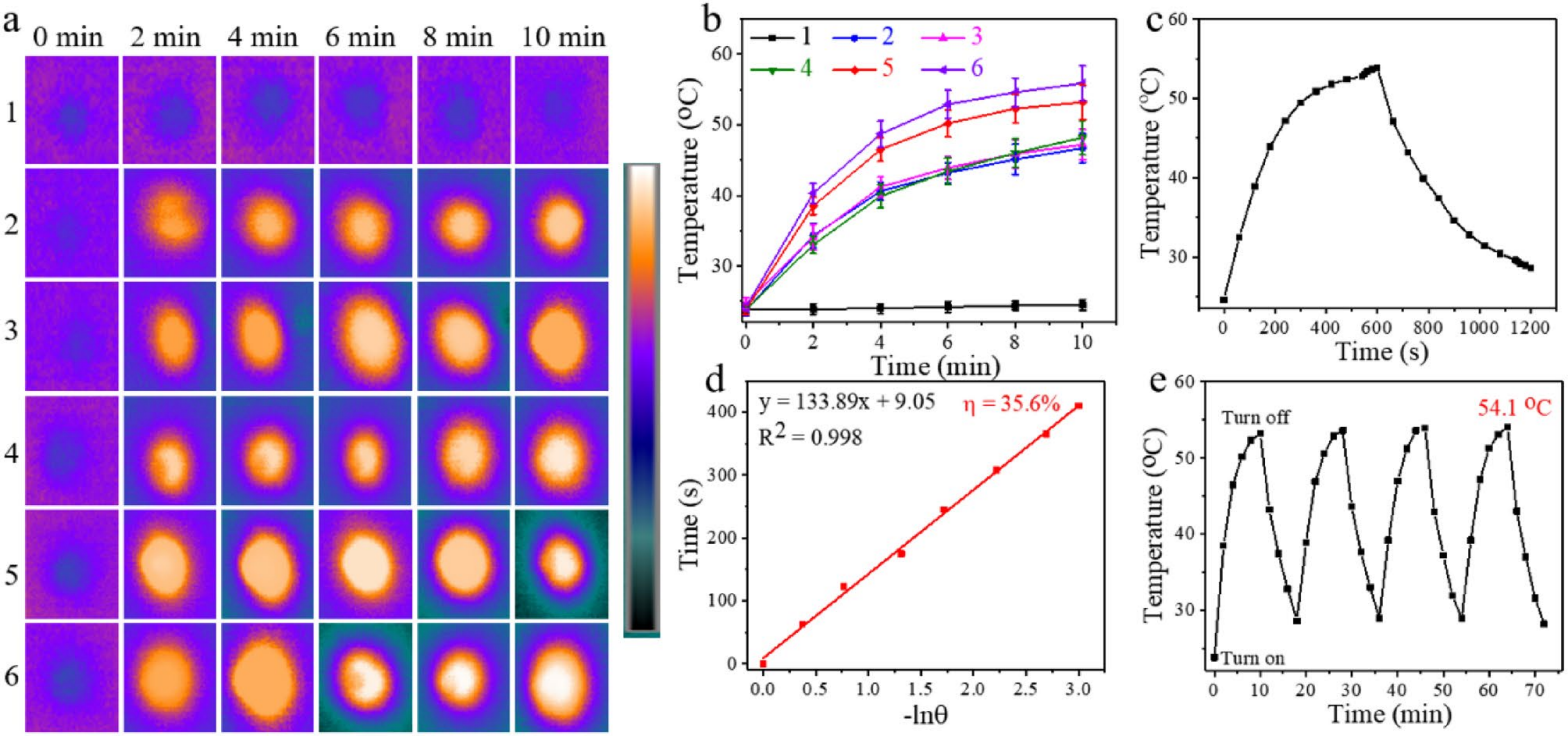
Photothermal Conversion Performance of PDA&DOX@LAC nanosystem. (**a**) Infrared thermal image showing the temperature distribution of PBS, PDA&DOX@LAC and polydopamine suspensions. (**b**) Temperature changes observed in PBS, PDA&DOX@LAC and polydopamine suspensions. (**c**) Photothermal response of the PDA&DOX@LAC dispersion solution over 600 s of irradiation followed by the cessation of irradiation. (**d**) Linear relationship between time and − lnθ during the cooling stage. (**e**) Temperature changes in the 200 μg/mL PDA&DOX@LAC dispersion solution under irradiation with an 808 nm NIR irradiation (2.0 W/cm^2^) for four on/off cycles. Legend: 1: PBS, 2: PDA&DOX@LAC (100 μg/mL, 2.0 W/cm^2^), 3: PDA&DOX@LAC (200 μg/mL, 1.5 W/cm^2^), 4: PDA&DOX@LAC (200 μg/mL, 1.75 W/cm^2^), 5: PDA&DOX@LAC (200 μg/mL, 2.0 W/cm^2^), 6: polydopamine (200 μg/mL, 2.0 W/cm^2^). Each sample was repeated three times.

The bacterial suspension of the PDA&DOX@LAC nanosystem exhibits absorbance in the near-infrared (NIR) region, which indicates polydopamine nanoparticles demonstrate excellent photothermal conversion properties. This prompted us to further evaluate the photothermal conversion performance of PDA&DOX@LAC. Therefore, bacterial suspensions of different concentrations of PDA&DOX@LAC nanosystem (100 µg/mL and 200 µg/mL) were subjected to irradiation under different power densities of NIR light (1.5 W/cm^2^, 1.75 W/cm^2^, and 2.0 W/cm^2^) for 10 min. As shown in Figs. 4a and 4b, the photothermal conversion performance of the PDA&DOX@LAC nanosystem is positively correlated with time, concentration and power density. When the concentration of PDA&DOX@LAC nanosystem was 200 µg/mL and irradiated with 2.0 W/cm^2^ NIR light for 10 min, the temperature of the PDA&DOX@LAC suspension could increase to 53.2 ℃, with minimal change compared to the temperature of polydopamine nanoparticles (55.9 ℃) under the same conditions. Furthermore, the photothermal conversion efficiency of the PDA&DOX@LAC nanosystem remained at 35.6% (Fig. 4c and 4d), which is not much different from the photothermal conversion rate of the pure polydopamine nanoparticles of 40%^55^. Thus, PDA&DOX@LAC exhibits superior photothermal conversion performance that is suitable for photothermal therapy of tumors. To further validate the photothermal stability of the PDA&DOX@LAC nanosystem for repeated irradiation, the 200 µg/mL PDA&DOX@LAC suspension was subjected to 2.0 W/cm^2^ NIR irradiation for 10 min and then the near infrared excitation was turned off for 10 min. This cycle repeated four times and temperature changes were recorded by an infrared thermal imaging instrument (recorded every 2 min). As shown in Fig. 4e, after four cycles of irradiation, the temperature of the PDA&DOX@LAC suspension remained at 54.1 °C, with a slight increase in temperature after each cycle, which was attributed to water evaporation caused by temperature rise. Therefore, the PDA&DOX@LAC nanoplatform prepared in this study demonstrates superior photothermal conversion performance and can withstand multiple repeated irradiation.

### Generation of ROS inside and outside PDA-DOX @ LAC cells stimulated by near infrared spectroscopy

**Figure 5:**
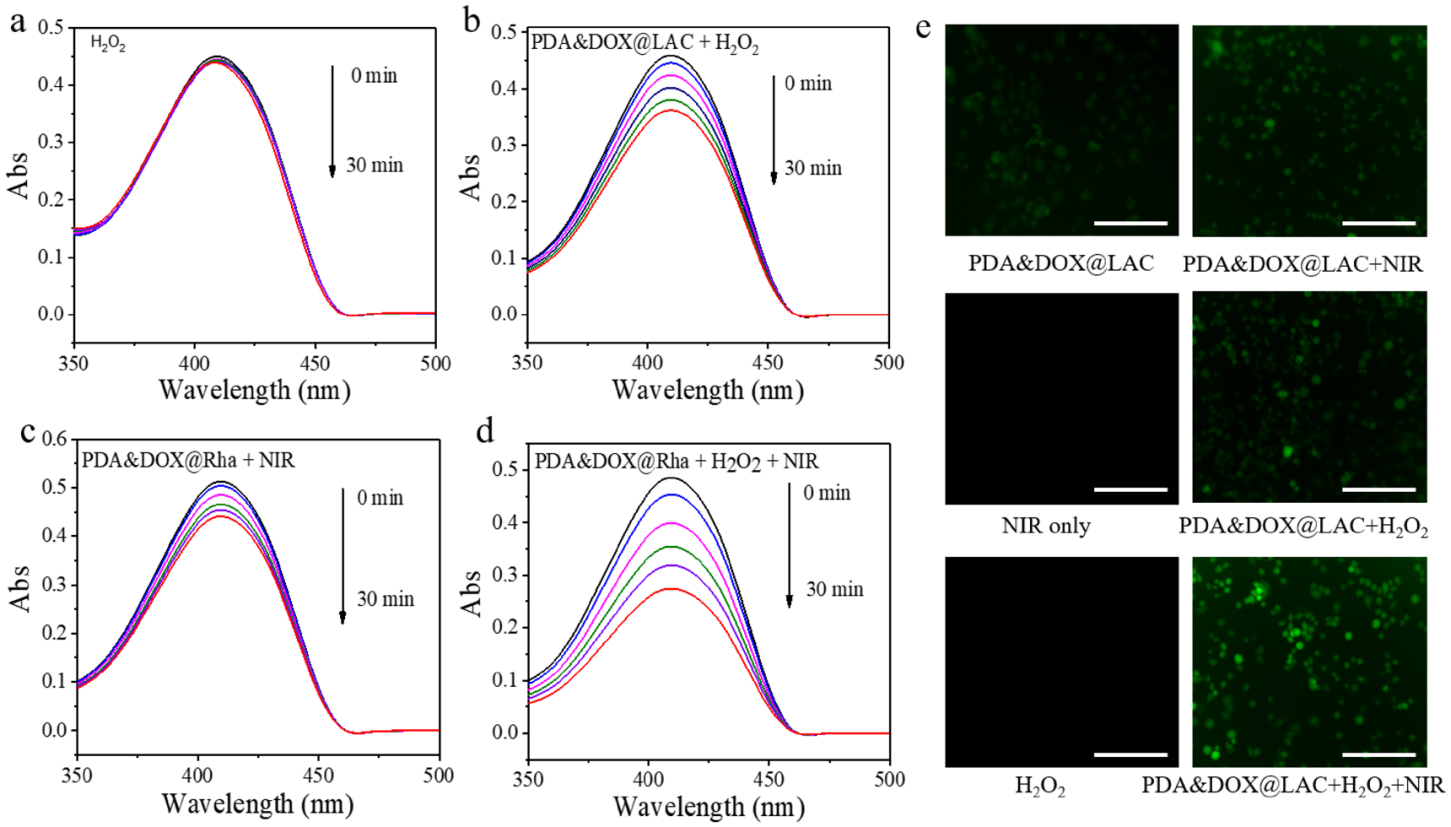
UV–vis absorption of DPBF in H_2_O_2_ (**a**), PDA&DOX@LAC + H_2_O_2_ (**b**), PDA&DOX@LAC + NIR (**c**) and PDA&DOX@LAC + NIR (**d**) at different time points. (**e**) CLSM images of DCF in 4T1 cells with PDA&DOX@LAC, PDA&DOX@LAC + NIR, NIR only, PDA&DOX@LAC + H_2_O_2_, H_2_O_2_, and PDA&DOX@LAC + H_2_O_2_ + NIR (6). Scale bars: 400 μm. Each sample was repeated three times.

The enzyme-like property and photothermal response property of PDA&DOX@LAC nanosystem prompted us to further explore its ability to generate reactive oxygen species (ROS) under the stimulation of NIR for photodynamic therapy (PDT) of tumors. As a typical extracellular active oxygen probe, DPBF can be oxidized to an oxidation product (O-dibenzoylbenzene) in the presence of ROS, resulting in the decrease of its UV absorption values^59^. To explore whether PDA&DOX@LAC could produce ROS under NIR irradiation, DPBF was respectively immersed in solutions of H_2_O_2_, PDA&DOX@LAC + H_2_O_2_, PDA&DOX@LAC + NIR, and PDA&DOX@LAC + H_2_O_2_ + NIR (Fig. 5a–d). Compared with the H_2_O_2_ group in the control group, the UV absorption values of DPBF in other groups decreased to some extent over time. It was mainly attributed to the Fenton reaction between PDA&DOX@LAC and H_2_O_2_, which generated hydroxyl radicals and caused the decrease of absorption value of DPBF. PDA&DOX@LAC produced a large amount of heat after NIR irradiation, which was then transferred to the oxygen in the surrounding tissue. The molecular oxygen transited to the ground state oxygen, causing the decrease of UV absorption values DPBF. However, after H_2_O_2_ was added into PDA&DOX@LAC solution and exposed under NIR for 10 min, the UV absorption values of DPBF exhibited a more significant decrease, indicating that Fenton reaction occurred after PDA&DOX@LAC was dispersed in H_2_O_2_ solution and resulted in the generation of hydroxyl free radicals and a large amount of oxygen. After NIR irradiation, more ^1^O_2_ generated.

PDA&DOX@LAC produced large amounts of ROS after extracellular stimulation with H_2_O_2_ and NIR, which prompted us to further evaluate the production of ROS in cells. DCFH-DA, as a cell permeability dye, has no fluorescence itself. Once it enters cells, it is easily hydrolyzed by cell esterase into DCFH, which is then rapidly oxidized by ROS to DCF with a strong green fluorescence product. Therefore, DCFH-DA was co-incubated with 4T1 cells to test whether PDA&DOX@LAC could produce ROS in 4T1 cells. As shown in Fig. 5e, 4T1 cells were randomly divided into 6 groups as PDA&DOX@LAC, PDA&DOX@LAC + NIR, NIR Only, PDA&DOX@LAC + H_2_O_2_, H_2_O_2_, and PDA&DOX@LAC + H_2_O_2_ + NIR. It is found by fluorescence microscope that the green fluorescence signal could be clearly observed when the 4T1 cells were incubated with PDA&DOX@LAC and irradiated with NIR for 10 min. In addition, after adding 30 µM H_2_O_2_ into the 4T1 cell culture medium and placing it under NIR irradiation for 10 min, the green fluorescence signal was the strongest compared with the control group. The main reason is that H_2_O_2_ reacts with PDA&DOX@LAC to produce more oxygen and a large amount of ^1^O_2_ is produced after NIR irradiation, which leads to the enhancement of green fluorescence signal. However, in other treatment groups like the H_2_O_2_ group and the PDA&DOX@LAC group, the green fluorescence signal could hardly be detected, mainly due to the small production of ROS in 4T1 cells, which is consistent with that result of in vitro DPBF detection of ROS. Therefore, PDA&DOX@LAC could produce a large amount of ROS under the excitation of NIR when there was sufficient oxygen around the tissue. Thus PDA&DOX@LAC can be used as an ideal material for PDT treatment.

### The killing effect of PDA&DOX@LAC on cells

**Figure 6:**
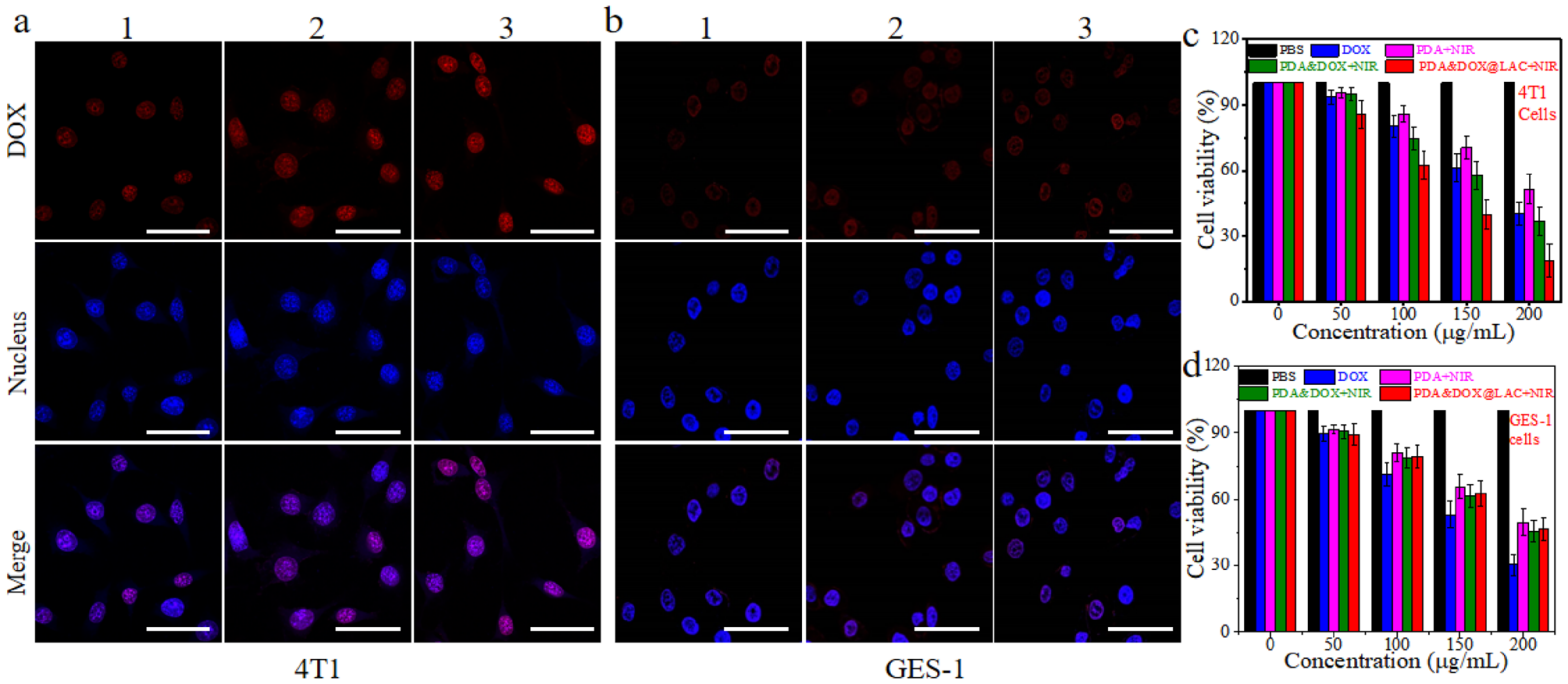
CLSM images of (**a**) 4T1 and (**b**) GES-1 cells incubated with PDA&DOX@LAC for 12 h and exposed under different power of NIR (1.5 W/cm^2^, 1.75 W/cm^2^ and 2.0 W/cm^2^). Scale bars: 30 μm. Viability of (**c**) 4T1 cells and (**d**) GES-1 cells upon treatment with PBS, DOX, PDA + NIR, PDA&DOX + NIR and DA&DOX@LAC + NIR. 1: 1.5 W/cm^2^ (10 min), 2: 1.75 W/cm^2^ (10 min), 3: 2.0 W/cm^2^ (10 min).

PDA&DOX@LAC has superior environmental response performance and the research showed that with the increase of temperature, it can effectively enhance the permeability of cell membrane and thus enhance the uptake of drugs by cells and enhance the killing performance of cells^29,30^. Therefore, 200 µg/mL PDA&DOX@LAC were incubated with 4T1 cells and GES-1 cells for 12 h respectively and then treated with NIR (1.5, 1.75 and 2.0 W/cm^2^) with different powers. The results of confocal microscopy showed that strong fluorescence signals could be detected in 4T1 cells and the fluorescence signal of DOX was also becoming stronger with the gradual increase of NIR power. It was mainly because the simulated tumor microenvironment constructed by 4T1 cells triggers the release of DOX from the surface of PDA&DOX@LAC. Subsequently, NIR causes the temperature to rise, which increases the permeability of 4T1 cells and promotes the uptake of DOX by 4T1 cells. (Fig. 6a). However, the fluorescence signal of DOX in GES-1 cells did not become stronger with the increase in NIR power (Fig. 6b). It is mainly due to the fact that the normal tissue microenvironment of GES-1 cells can't release DOX from the surface of PDA&DOX@LAC, which causes that GES-1 cells can't absorb DOX and its fluorescence signal doesn't get stronger with the increase of NIR power.

Thanks to the effective environmental responsiveness of drug release exhibited by PDA&DOX@LAC and the efficient drug absorption capability of tumor cells, we have delved deeper into its cytotoxic effects on tumor cells. Specifically, the viability of both GES-1 and 4T1 cells was assessed through CCK-8 experiments to gauge the antitumor efficacy of the chemo-photothermal synergistic therapy mediated by PDA&DOX@LAC. As depicted in Figs. 6c and 6d, the escalation in PDA&DOX@LAC concentration corresponded to a gradual decline in the cell viability of both GES-1 and 4T1 cells. Upon exposed to NIR (10 min, 2.0 W/cm^2^) under identical concentrations and conditions, the viability of 4T1 cells notably plummeted compared to that of GES-1 cells. Specifically, at a concentration of 200 μg/mL of PDA&DOX@LAC, the cell viability of GES-1 cells remained approximately at 50%, whereas that of 4T1 cells dipped below 20%. These findings underscored the potent cytotoxicity of the chemo-photothermal synergistic therapy mediated by PDA&DOX@LAC against tumor cells, which was primarily attributed to the effective tumor microenvironment-driven DOX release and subsequent enhancement of 4T1 cell permeability facilitated by photothermal therapy, thereby augmenting DOX absorption^60^. In addition, when DOX and PDA&DOX@LAC were in the same concentration, the killing effect of DOX on GES-1 cells was significantly higher than that of PDA&DOX@LAC. Furthermore, when DOX and PDA&DOX@LAC were administered at equivalent concentrations, the cytotoxic efficacy of DOX against GES-1 cells significantly surpassed that of PDA&DOX@LAC. Hence, PDA&DOX@LAC achieves a multifaceted approach including chemotherapy, photothermal therapy, and PDT, supplemented by near infrared radiation, effectively eradicating tumor cells while mitigating DOX-induced damage to normal cells.

### Antitumor effect of PDA&DOX@LAC in vivo

**Figure 7:**
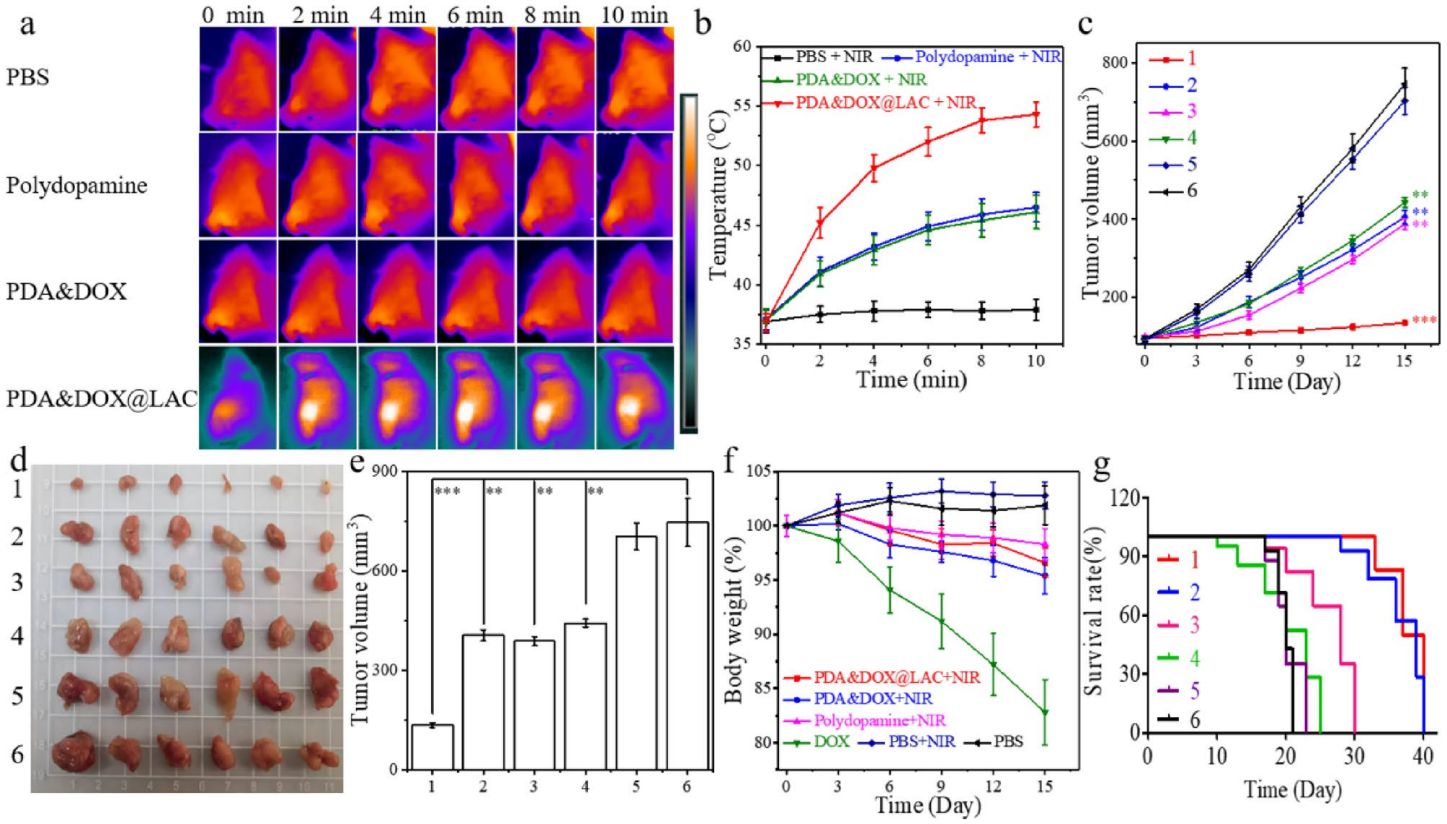
(**a**) Infrared thermal images of mice at varied time points of different treatments under NIR irradiation with the power of 2.0 W/cm^2^ for 10 min. (**b**) Temperature change curves at tumor sites of mice in different groups upon NIR irradiation. (**c**) Average tumor growth curves of 4T1 tumor-bearing mice after 15 d of different treatments (n = 6/group). (**d**) Photographs of the tumors and (**e**) average weights of tumor tissues on day 15 after the last treatment. (**f**) Body weight of mice in different groups during treatment. (**g**) The survival curves of mice in the different treatment groups. 1: PBS, 2: PBS + NIR, 3: Polydopamine + NIR, 4: DOX, 5: PDA&DOX + NIR, 6: PDA&DOX@LAC + NIR.

Based on the excellent photothermal conversion performance of the PDA&DOX@LAC nanosystem in vitro, we further evaluated its aggregation performance in tumor tissues of 4T1 tumor-bearing mice to examine its antitumor effect in vivo. To begin with PBS, polydopamine nanoparticles, PDA&DOX, and PDA&DOX@LAC were injected into mice with 4T1 xenografts through their tail vein. After 24 h, the 4T1 tumor-bearing mice in each group were irradiated with NIR (2.0W/cm^2^) for 10 min and the changes in tumor tissue temperature in each group were recorded by infrared thermal imaging (Fig. 7a and Fig. 7b). When 4T1 tumor-bearing mice were treated with PDA&DOX@LAC, the temperature of tumor tissue increased to 54.3 ℃. After polydopamine and PDA&DOX nanoparticles treatment, the temperature of tumor tissue only increased to 46.5 ℃ and 46.1 ℃. To sum up, the PDA&DOX@LAC nanosystem increased the targeted delivery of PDA&DOX@LAC to tumor tissues with the help of the anaerobic feature of LAC.

Then we further evaluated PDA&DOX@LAC nanosystem antitumor performance. When the 4T1 xenografts grew to ~ 100 mm^3^, 4T1 tumor-bearing mice were randomly divided into six groups (n = 6/group) and treated with PBS, PBS + NIR, polydopamine + NIR, DOX, PDA&DOX + NIR and PDA&DOX@LAC + NIR respectively. The changes in tumor tissues and body weight of these mice were recorded every day. Figure 7c showed that synergistic treatment of PDA&DOX@LAC + NIR effectively inhibits the growth of xenografts in mice. After 15 d of treatment, the average size of tumor tissues was only 135mm^3^ in the PDA&DOX@LAC + NIR group, compared to 405.6 mm^3^, 442.6 mm^3^, and 387.6 mm^3^ in the polydopamine + NIR, DOX and PDA&DOX + NIR groups respectively. In addition, NIR irradiation alone did not significantly inhibit the growth of xenografts in mice (Fig. 7d and Fig. 7e). It was found that the body weight of the mice decreased rapidly after treatment with DOX and they even became depressed (Fig. 7f). In groups of polydopamine + NIR, PDA&DOX + NIR and PDA&DOX@LAC + NIR, only a slight change in the body weight of tumor-bearing mice can be observed and the life cycle of the mice was longer than that in the DOX treatment group. In particular, after 40 d of PDA&DOX@LAC + NIR synergistic therapy, the survival rate of mice in this group remained around 30%, while mice in all the other treatment groups had already died (Fig. 7g). In a word, PDA&DOX@LAC + NIR synergistic treatment could not only effectively inhibit the growth of xenografts in mice, but also greatly reduce the toxic and side effects of DOX and extend their life.

### Analysis of antitumor mechanism of PDA&DOX@LAC in vivo

**Figure 8:**
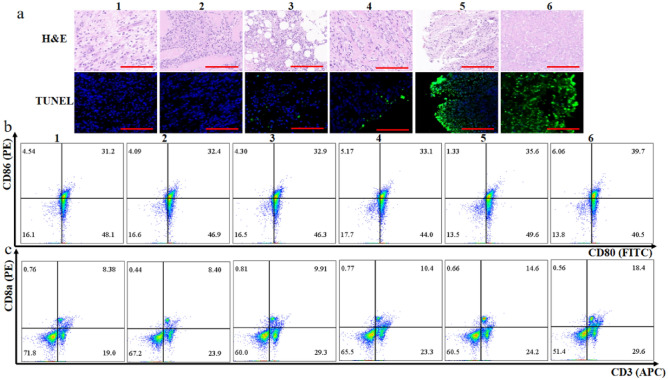
(**a**) H&E staining (up) and TUNEL staining (down) of tumor slices collected from 4T1 tumor-bearing mice after different treatments. Scale bars: 100 μm. Flow cytometry plots of (**b**) dendritic cells maturation and (**c**) CD3^+^/CD8a^+^ T cells extracted from tumor tissues after different treatments. 1: PBS, 2: PBS + NIR, 3: polydopamine + NIR, 4: DOX, 5: PDA&DOX + NIR, 6: PDA&DOX@LAC + NIR.

Given that PDA&DOX@LAC + NIR synergistic therapy effectively inhibits the growth of mouse xenografts, we further analyzed its antitumor mechanism. After 15 d of treatment, the tumor tissues of mice in each treatment group were collected for TUNEL and H&E staining and the necrosis as well as apoptosis of tumor cells in the tumor tissues were analyzed. As shown in Fig. 8a, compared with the polydopamine + NIR, DOX, PDA&DOX + NIR, and PDA&DOX@LAC + NIR treatment groups, no obvious necrosis and apoptosis of tumor cells were observed either in the PBS or in PBS + NIR treatment groups. H&E staining results indicated that chemo-photothermal synergistic therapy of PDA&DOX@LAC was highly destructive to tumor cells, which is featured by vacuolation, karyopycnosis, and karyolysis. In addition, TUNEL staining showed that a green fluorescence signal was only detected on the surface of tumor tissue in polydopamine + NIR and PDA&DOX + NIR treatment groups while the green fluorescence signal of the tumor tissue was weaker after DOX treatment. However, after chemo-photothermal synergistic therapy in PDA&DOX@LAC + NIR group, a large number of green fluorescence signals could be found. To conclude, the PDA&DOX@LAC nanosystem could effectively transport chemotherapeutic drugs and photothermal carriers to the deep part of tumor tissue to release DOX and kill tumor cells in the deep part of tumor tissue because LAC easily survive in anoxic and hypoxic environments. And then, apoptotic tumor cells release tumor-associated antigens, thereby promoting the immune response of the body. Therefore, tumor cells from each treatment group were collected to analyze the immune cells in tumor tissues, which helps to explore the immune regulation in the nanosystem PDA&DOX@LAC chemo-photothermal synergistic therapy. As shown in Fig. 8b, the proportion of dendritic cells (PDA&DOX@LAC) in the PDA&DOX@LAC treatment group exposed to NIR was 39.7%, which was higher than that in other treatment groups (PBS, PBS + NIR, polydopamine + NIR, DOX, and PDA&DOX + NIR). This is due to the efficacy of PDA&DOX@LAC in killing tumor cells and promoting the release of tumor-associated antigens after the chemophotothermal synergistic treatment of PDA&DOX@LAC, thus increasing the dendritic cells’ absorption of it and eventually leading to the maturation of dendritic cells. However, cytotoxic T lymphocytes (CD8^+^ T cells) play an important role in the antitumor immune response by being activated by tumor-derived antigens and then directly killing tumor cells. Therefore, the expression of CD8^+^ T cells in tumor tissue was analyzed. The percentages of CD8^+^ T cells in the PBS, PBS + NIR, polydopamine + NIR, DOX and PDA&DOX + NIR treatment groups were 8.38%, 8.4%, 9.91%, 10.4%, and 14.6% respectively (Fig. 8c). However, chemo-photothermal synergistic therapy of PDA&DOX@LAC resulted in the highest percentage of CD8^+^ T cells among all groups, which was 18.4%. These results indicated that the PDA&DOX@LAC nanosystem, as a potential in situ vaccine, could significantly activate the immune system, promote dendritic cells maturation and CD8^+^ T cell activation and then kill tumor cells and inhibit the growth of tumors.

### PDA&DOX@LAC biocompatibility

**Figure 9:**
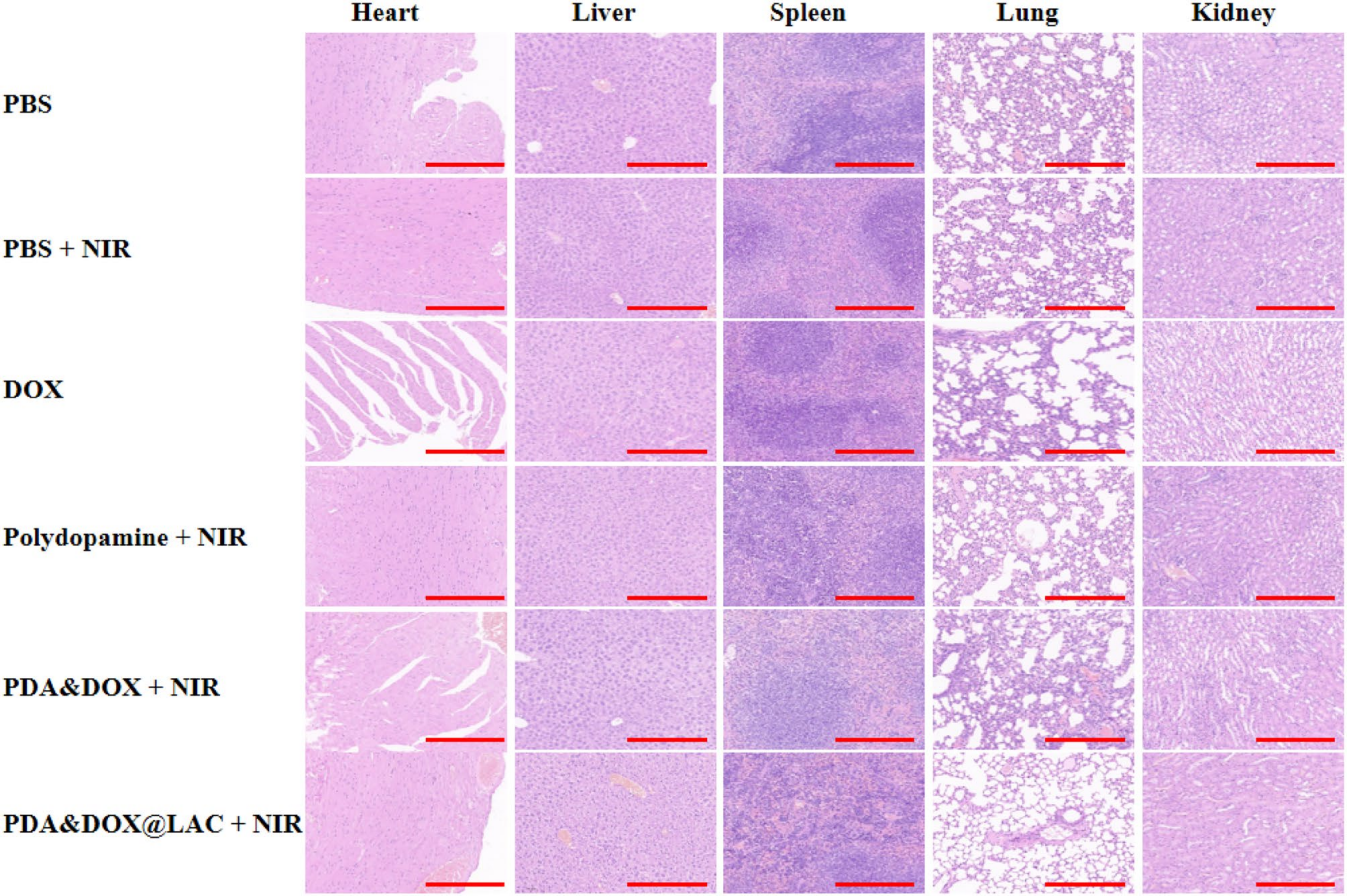
H&E staining of various organs collected from 4T1 tumor-bearing mice after different treatments of PBS, PBS + NIR, DOX, polydopamine + NIR, PDA&DOX + NIR and PDA&DOX@LAC + NIR for 15 days. Scale bars: 100 μm.

**Figure 10:**
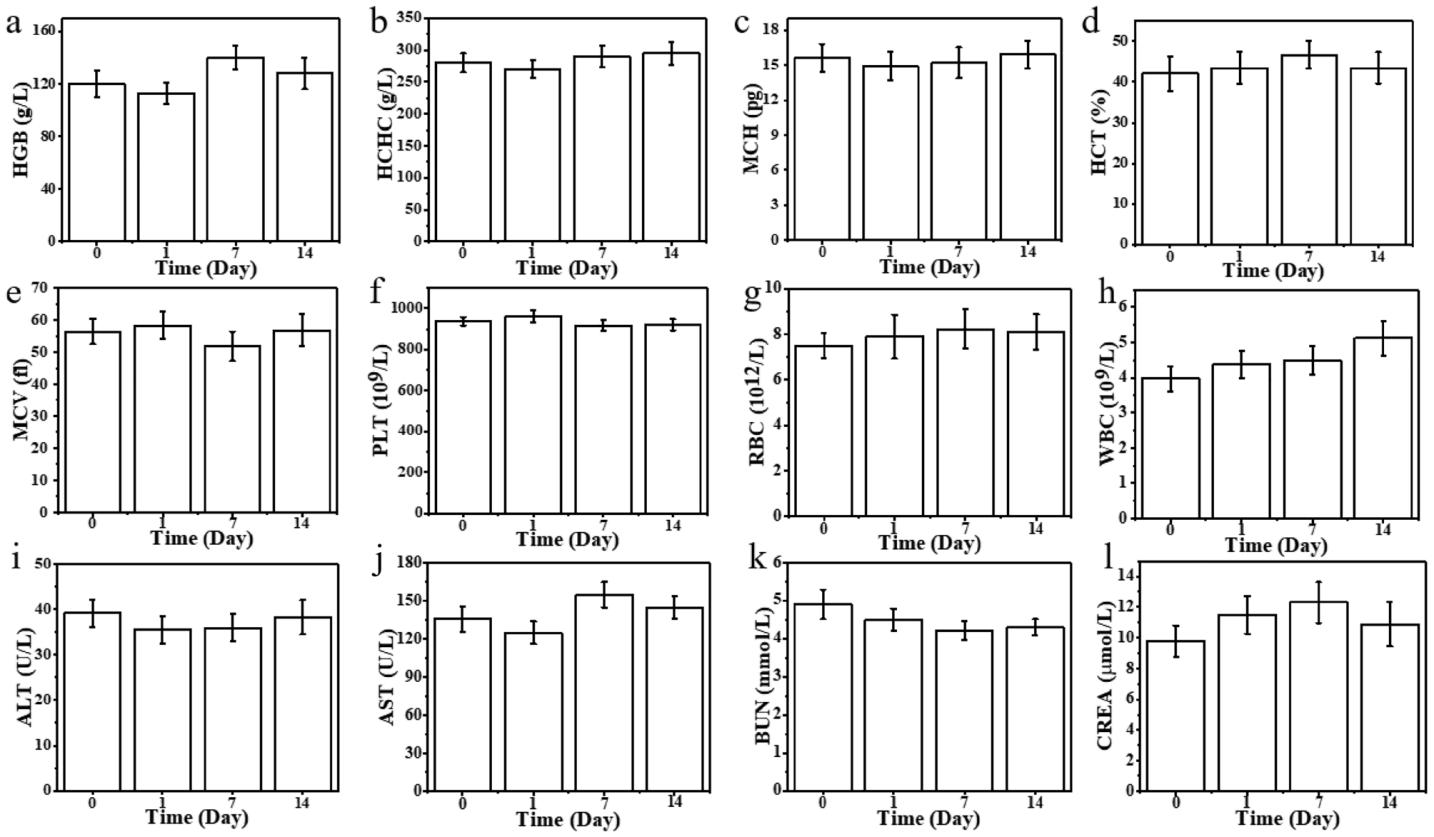
The blood tests (**a**–**h**) and the serum biochemistry index (**i**–**l**) of the mice on the 0th, 1st, 7th, and 14th day after the treatment of PDA&DOX@LAC.

Chemo-photothermal synergistic therapy of the PDA&DOX@LAC nanosystem exhibits superior antitumor effect, so it is used for the treatment of mouse xenografts and future clinical application. It requires low toxicity or even non-toxicity in vivo so the biocompatibility should be analyzed through animal experiments. The biocompatibility of PDA&DOX@LAC in vivo was analyzed by H&E staining during the treatment of tumor-bearing mice. After the treatment, H&E staining was performed on the main organs (heart, liver, spleen, lung, and kidney) of mice in each treatment group to compare with the control group. It was found that when the mice were injected with PDA&DOX@LAC nanosystem for 15 d, no significant inflammation or tissue damage was found in the pathological sections. Moreover, the PDA&DOX@LAC nanosystem could effectively alleviate the toxic and side effects of DOX in the tumor treatment process and especially reduce the damage of DOX to heart tissues (Fig. 9). Then, blood samples of the PDA&DOX@LAC + NIR treatment group were collected for routine blood tests and serum biochemical indicators tests. As shown in Fig. 10, blood was drawn from 4T1 tumor-bearing mice on days 0, 1, 7, and 14 of PDA&DOX@LAC + NIR treatment, respectively. The results showed that there were no obvious changes in routine and biochemical blood test results during the PDA&DOX@LAC + NIR treatment. The above results indicated that PDA&DOX@LAC exhibits excellent biocompatibility as a drug carrier during tumor treatment.

## Conclusion

In conclusion, the researches detailed above have led to the development of a live bacterial drug carrier endowed with enzyme-like properties, which are suitable for targeted tumor synergistic therapy. The PDA&DOX@LAC nanosystem employed exhibits notable advantages: (1) mitigating the toxicities and adverse effects of DOX on normal tissues in vivo during tumor intervention; (2) facilitating targeted delivery to tumor sites and effective tumor cell eradication through LAC's adaptability within anoxic environments; (3) harnessing the catalase property of the PDA&DOX@LAC nanosystem to ameliorate tumor tissue hypoxia and thereby augmenting oxygen availability for photodynamic therapy; (4) enabling the synergistic photothermal therapy of PTT and PDT via NIR laser activation in a single procedure; (5) fostering the generation of tumor-associated antigens and eliciting a robust anti-tumor immune response through the chemo-photothermal synergistic therapy mediated by the PDA&DOX@LAC nanosystem. Therefore, the results of in vivo and in vitro treatment emphasize the innovative and effective role of PDA and DOX LAC nano-system in promoting the platform of tumor collaborative treatment, thus furnishing a theoretical foundation and design paradigm for future iterations of tumor treatment modalities.

## Data Availability

The datasets supporting the results in this article are included within the article.
